# Effects of Physical Activity on Body Composition, Muscle Strength, and Physical Function in Old Age: Bibliometric and Meta-Analyses

**DOI:** 10.3390/healthcare12020197

**Published:** 2024-01-13

**Authors:** Yerim Choi, Daekyoo Kim, Seung Kyum Kim

**Affiliations:** 1 Convergence Institute of Biomedical Engineering and Biomaterials, Seoul National University of Science and Technology, Seoul 01811, Republic of Korea; ye8rim21@seoultech.ac.kr; 2 Department of Physical Education, Korea University, Seoul 02841, Republic of Korea; daekyookim@korea.ac.kr; 3 Department of Sports Science, Seoul National University of Science and Technology, Seoul 01811, Republic of Korea

**Keywords:** aging, physical activity, bibliometric analysis, meta-analysis, frailty, resistance training

## Abstract

Objectives: Accumulating evidence suggests that physical activity (PA) is an efficient intervention to maintain functional capabilities and mitigate physiological changes in the older population. However, an attempt has yet to be made to comprehensively investigate the published landscape on the subject. Methods: This study had two aims. The first aim was to perform a bibliometric analysis for two keywords, “aging” and “PA”, to analyze the research trend. Since “frailty” was the most noticeable co-occurring keyword with the two keywords, the second aim was to investigate the effects of PA, particularly, resistance training (RT), on frailty using a meta-analysis to provide a summary of the current evidence base. Results: The bibliometric analysis revealed that the number of publications on this research topic has gradually increased, highlighting the importance of understanding the role of PA in aging. The meta-analysis found that RT had significant beneficial effects on physical frailty factors, including handgrip strength, lower limb strength, balance, gait speed, and stair-climbing ability. Conclusion: These findings demonstrate that RT is an effective intervention for improving physical function in frail populations; thus, it has important implications for the development of PA programs for older adults with frailty. Future research is warranted to explore the optimal dose, frequency, and duration of RT programs for older adults, as well as the potential benefits of combining RT with other forms of PA, such as aerobic or balance exercises.

## 1. Introduction

Aging is a natural biological process that results in a progressive decline in physical and cognitive functions [1,2]. A significant consequence of aging is its association with physical inactivity or disuse, resulting in a decline in muscle mass, structure, and strength [3]. This leads to changes in the quantity and quality of skeletal muscles, which can worsen muscle weakness and disability in the older population [4]. Accordingly, physical disability affects a substantial proportion of older adults, with 44% of those aged 65 years or older experiencing physical weakness, thereby increasing the risk of impairments in activities of daily living by 54% [5].

As the global population ages, there is a growing focus among healthcare providers to understand and intervene in the factors that increase the risk of health and functional declines in older adults [6,7]. Frailty syndrome, a clinical state characterized by increased vulnerability to stressors, leading to negative health-related outcomes in individuals [8], represents instability and the risk of current or further loss of function [9]. The frequency of frailty syndrome increases with age and is more prevalent among individuals with disabilities, depression, hip fractures, and other comorbidities, such as cardiovascular disease and nervous system disorders [10]. Therefore, maintaining physical health and functional capacity in older adults is a critical public health concern, garnering significant attention in the context of healthy aging.

According to the WHO World report on aging and health [11], healthy aging encompasses all the mental and physical capacities that an individual possesses, including cognitive function, sensory function, vitality, motor function, and psychological well-being, with the central goal of preserving and optimizing intrinsic capacity. In particular, the report emphasizes physical activity (PA) as a key strategy to counteract or postpone decreases in intrinsic capacity and conditions like frailty, which is a geriatric syndrome resulting from the declines in multiple physiological systems and thus has become one of the biggest challenges in facilitating healthy aging [12]. PA can serve as a polypill that improves health-related quality of life and functional capabilities while mitigating the physiological changes and comorbidities associated with aging [13,14,15,16]. It is also a fundamental approach targeting age-related declines in physical function parameters, such as muscle strength, mobility, gait, and balance, which are major concerns in maintaining the intrinsic capacity of frail individuals [17,18]. Over the past few decades, studies exploring the role of PA as a determinant of successful aging in the health and functional status of older individuals have accumulated [19]. Nevertheless, efforts to synthesize findings, identify prominent trends, and identify research gaps in the accumulation are still absent. To complement this, we conducted this study with the following two aims, which are sequential but independent of each other.

The first aim of this study was to perform a quantitative bibliographic analysis, which is a methodology that uses statistical tools and techniques to analyze and interpret bibliographic data [20] and thus can be a valuable approach to understanding research trends in the research area [21,22], using two keywords: “aging” and “physical activity”. An analysis of keywords used by authors or the content of published papers can reveal present and potential future trends in a research area, and the use of bibliometric analysis is becoming more widespread in various research fields [20]. Specifically, the first aim focuses on using keyword network analysis to map the connections between two keywords and others and to identify the most noticeable research themes in the research topics having the two keywords. As a result of the first aim of the bibliometric analysis, we identified the most prominently growing keyword (herein, “frailty”) that co-occurred with aging and PA. Thus, as a follow-up, but independent research approach, our second aim was to conduct a meta-analysis of studies exploring the effects of PA (particularly, resistance training) on frailty in order to synthesize the findings of previous studies and provide a comprehensive summary of the current evidence base, focusing on the effects of resistance training (RT) on body composition, muscle strength, and physical function.

The findings of this study have several implications for researchers, healthcare professionals, and policymakers working to promote healthy aging with PA interventions. First, by identifying the most prominent research trends and gaps using bibliometric analysis, this study can guide future research in this area. Second, this study provides a comprehensive summary of the current evidence, informing the development of evidence-based practice guidelines for PA interventions in older adults. Therefore, this study raises awareness of the importance of PA as a key intervention for promoting healthy aging and highlights the need for further research and investment in this field.

## 2. Materials and Methods

### 2.1. Bibliometric Analysis Method

Articles containing the two keywords “aging” (including aged, old age, old adults, older adult, older people, senior, etc.) and “physical activity” (including exercise, resistance training, aerobic training, endurance training, and physical fitness) were searched in SCOPUS, Web of Science, and PubMed on 1 August 2023. Since there were only a few papers (n < 100) published before 1991, we only included articles published from January 1991 to July 2023 in the analysis.

**Figure 1:**
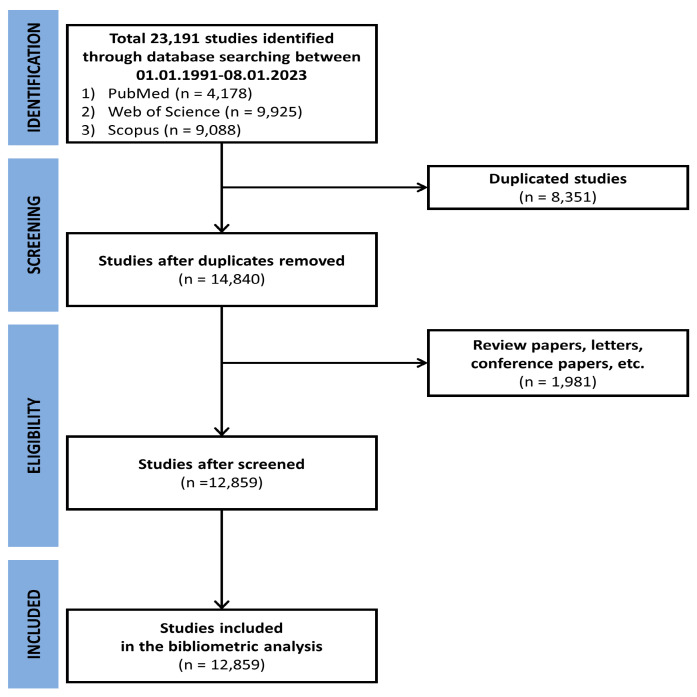
Flowchart of the bibliometric analysis selection process.

Data extraction initially selected 9088 papers from SCOPUS, 9925 from Web of Science, and 4178 from PubMed. After merging these papers and removing duplicates, 14,840 papers constituted the primary body of the literature. The collected studies were further selected by excluding duplicates and non-original research, and 12,859 publications met the inclusion criteria (Figure 1).

The keywords extracted from the final publications were reviewed by two researchers (YC and DK), where standardized similar words were examined to unify the terms among the evaluators. The keyword analysis and network visualization were performed using VOSviewer 1.6.18 [23].

### 2.2. Meta-Analysis Method

**Figure 2:**
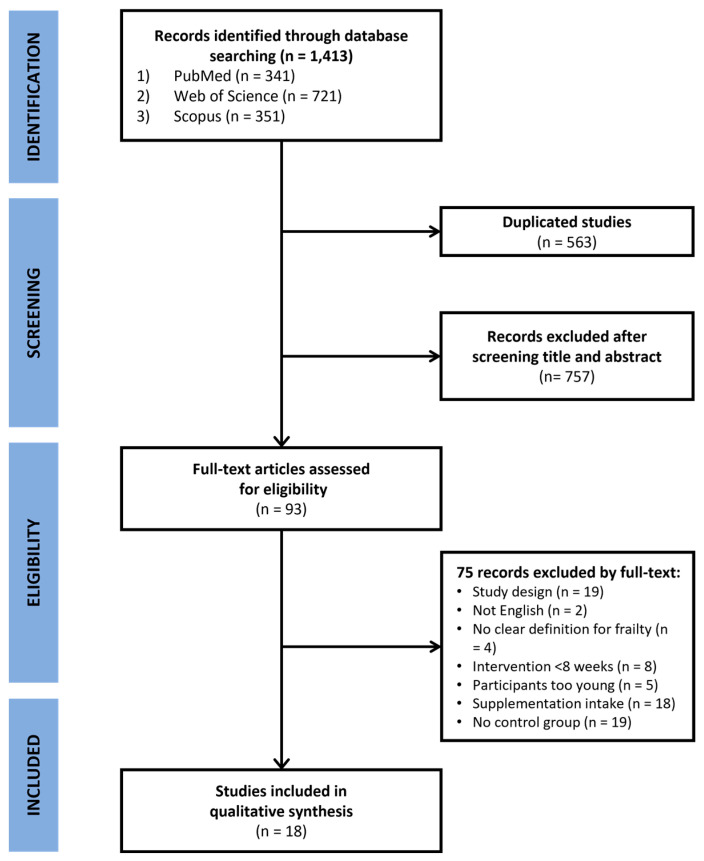
PRISMA flowchart for the study identification procedure of the meta-analysis.

Since the results from the bibliometric analysis indicated that “frailty” is the prominently growing co-occurring keyword with aging and PA research (see Table 1), we further conducted a meta-analysis for studies investigating the effect of PA on old frail individuals from January 1991 to July 2023 (the same period as the bibliometric analysis). In particular, we chose resistance training (RT) as a type of PA since previous studies demonstrated that RT solely appeared to be effective [24], and the effects of other types of exercise, such as aerobic and flexibility, on frailty are yet to be established [12]. The meta-analysis was conducted according to the Preferred Reporting Items for Systematic Reviews and Meta-Analysis (PRISMA) statement [25]. We used the PICO framework to conduct our search using the following criteria: (1) population: individuals aged 65 or older exhibiting pre-frailty or frailty without additional health conditions (e.g., diabetes, cancer, stroke, dementia, depression); (2) intervention: focus on RT, either alone or combined with other training components, targeting muscle mass, muscle strength, functional capacity, and fall incidence in frail older adults; (3) comparison: absence of RT, with studies featuring alternative control interventions, like home-based exercise or educational programs, also considered; (4) outcome: assessment of body composition (muscle mass, fat mass, BMI), muscle strength (handgrip strength, lower limb strength), and physical performance (balance, timed up and go test, gait speed); and (5) study design: limited to randomized controlled trials. We used the following keywords: (aging OR aged OR older people OR elderly OR old age OR older adult OR senior) AND (frailty OR frail elderly OR frail OR pre-frail OR pre-frail elderly OR frail older adults OR frail older) AND (resistance training OR resistance exercise OR weightlifting OR strengthening programs OR strength training OR strength exercise OR weight training OR weight exercise). Electronic database searches were performed on 1 August 2023. After identifying 1413 potential studies with an initial search using three search engines (341 articles from PubMed, 721 articles from Web of Science, and 351 articles from SCOPUS), 563 duplicate studies were excluded. We excluded 832 studies based on the inclusion and exclusion criteria. The inclusion criteria were: (1) studies that recruited older adults (age ≥65) with pre-frailty or frailty but without comorbid conditions (e.g., diabetes, cancer, stroke, dementia, and depression); (2) studies that estimated the quantitative changes in the outcomes of interest (body composition, muscular strength, balance, and agility); (3) RT interventions that lasted for at least 8 weeks since this is the minimally recommended intervention period to improve muscle strength [26], and (4) manuscripts published in English. The exclusion criteria were as follows: (1) cognitive or social frailty; (2) disability (e.g., advanced disability in performing daily activities, dementia, or end-stage disease); and (3) the use of supplements. Finally, the remaining 18 studies [27,28,29,30,31,32,33,34,35,36,37,38,39,40,41,42,43,44] with only randomized controlled trial designs were selected for the meta-analysis, as shown in the PRISMA flow diagram (Figure 2).

#### 2.2.1. Data Extraction

Two authors (YC and DK) independently extracted the following variables from the included studies: (1) study characteristics (year of publication, geographical area) and the sample (size, sex, and age); (2) program description for the training and control groups; (3) main outcomes of interest; and (4) overall effect of the outcome of interest. We gathered the group size and mean differences in the outcomes of interest for both groups (intervention and control) with a 95% confidence interval (CI) or standard deviation (SD) for quantitative analyses (meta-analyses). An excel document created specifically for the meta-analyses contained all the tabulated data. The authors cross-checked each other’s coding sheets, and disagreements were settled through discussion and consensus.

#### 2.2.2. Statistical Analysis

A standardized mean difference (SMD), a point estimate of the treatment effect, with a 95% CI was utilized to describe the RT effect. As a measure of effect size to explore differences between RT and control conditions, Hedges’ g was calculated based on the quantitative data [45], including the sample size, mean, and standard deviation of both groups at baseline test and post-test. Values of 0.2–0.49, 0.5–0.79, and >0.79, respectively, were used to identify small, moderate, and high effect sizes [46]. Heterogeneity was assessed utilizing the I-squared (I^2^) index, which measures between-study heterogeneity. In order to represent low, moderate, and high heterogeneity, respectively, values of I^2^ greater than 25%, 50%, and 75% were used [47]. Due to the wide range of studies included in the meta-analyses, random-effect models were applied. This model was used to calculate the overall or mean effect size under the assumption that the samples originate from populations with varying effect sizes and the genuine effect diverges across studies [48]. Statistical significance was set at *p* < 0.05. Meta (version 2.0) and metafor (version 2.0-0) in R version 3.4.4 (The R Foundation for Statistical Computing, Vienna, Austria) were utilized for all analyses.

#### 2.2.3. Quality Assessment

The methodological quality of each study was evaluated using the Physiotherapy Evidence Database (PEDro) scale (0–10), which can assess randomized controlled trials [49]. PEDro scale has four categories: “poor” (scores 0–3), “fair” (scores 4–5), “good” (scores 6–8), and “excellent” (scores 9–10). Trials with scores less than 4 were eliminated, while higher PEDro scores denoted higher quality.

## 3. Results

### 3.1. Bibliometric Analysis

#### 3.1.1. Trend of Publication Year-Wise

**Figure 3:**
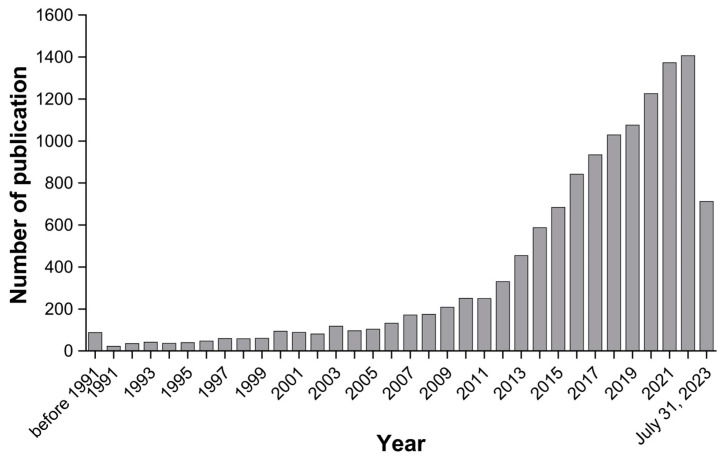
The number of publications having the two keywords “aging” and “physical activity”.

The number of articles published per year that included the two keywords is shown in Figure 3. The number of publications has gradually increased over time. Although this increasing trend could also be associated with a general increase in the number of researchers and articles published on any subject every year, it is to be noted that 6029 out of 12,859 articles were published in the past six years (2017–2023). This demonstrates an increasing trend in aging and PA, which is becoming increasingly important on a global scale.

#### 3.1.2. Keyword Analysis

**Figure 4:**
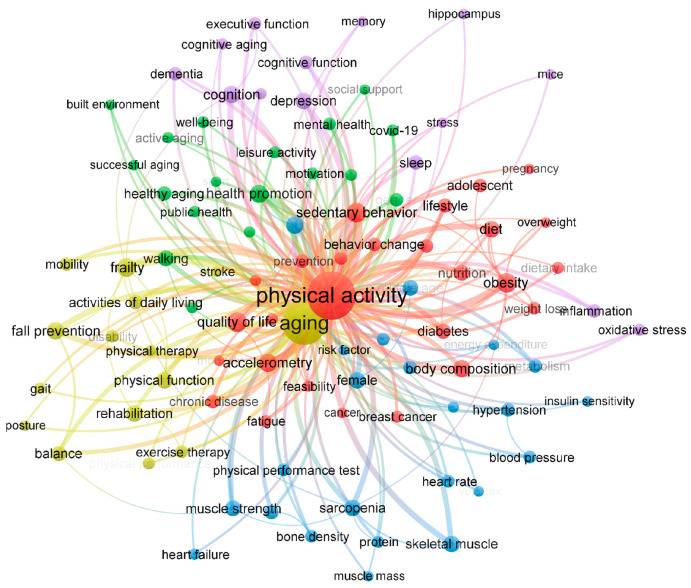
The keyword network from original articles having the two keywords “aging” and “physical activity” and published between January 1991 and July 2023 (appeared ≥60 times). The similarity between two keywords is proportional to the number of simultaneous occurrences, placing words with high similarity close together. The size of the circles representing keywords increases with the relevance of the keywords (total link strength), and the distinctive colors of the circles and lines mean the difference in modularity.

We analyzed 15,494 author keywords from 12,859 articles including the two keywords. Figure 4 visually represents the network of these keywords and illustrates the strength of their associations based on the number of articles in which they appeared together [50]. The similarity between two words is proportional to the number of simultaneous occurrences, placing words with high similarity close to each other. The size of the circles representing keywords increases with the relevance of these words, and the distinctive colors of the circles and lines indicate differences in modularity. The thicker the line connecting the two keywords, the stronger their association [51]. After trimming for a minimum of 60 co-occurrences, 98 keywords were shortlisted.

Among these keywords, “sedentary behavior”, “quality of life”, “obesity”, and “frailty” emerged as the most frequently co-occurring terms. They were also closely associated with other keywords, such as “balance”, “activities of daily living”, “fall prevention”, “muscle strength”, “walking”, and “health promotion”.

Furthermore, the results of the bibliometric analysis revealed three distinct clusters of co-occurring keywords, each representing a set of closely related terms. Cluster 1 included words like “frailty”, “fall prevention”, “rehabilitation”, and “balance”, which are directly associated with physical function in old individuals. Cluster 2, on the other hand, contained keywords including “sedentary behavior”, “obesity”, “diet”, and “body composition”. This suggests a focus in previous studies on the importance of lifestyle and habits in aging and PA research. Lastly, Cluster 3 consisted of terms, such as “depression”, “dementia”, “cognitive function”, and “memory”, demonstrating a research emphasis on the relationships among cognitive aspects, aging, and PA.

#### 3.1.3. Trends in Keywords by Time

To classify research trends by era, keywords were grouped by decades (1991–2000 vs. 2001–2010 vs. 2011–2023). It was observed that 562, 1720, and 10,919 manuscripts were published in the first, second, and third decades, respectively, which also supports the finding that the number of publications on this research topic has gradually increased.

Table 1 shows the top 30 keywords that appeared most frequently with the two searched keywords for each decade. The number of occurrences and their total link strength with other keywords were calculated for all keywords. In the first decade, the keywords “body composition”, “activities of daily living”, “health promotion”, and “smoking”, were noticed, suggesting that the effect of aging and PA on lifestyle and daily living were the primary focus of research during this period. In addition, the keywords “female” and “gender” were highly ranked, suggesting that gender differences in aging and PA were also highlighted topics during the period. The frequently co-occurring keywords in the second decade were similar to those in the first decade. Of note, the keywords associated with diseases, such as “frailty”, “depression”, “sarcopenia”, and “cognition” began to appear in earnest during this period. Notably in the past decade, the keyword “sedentary behavior” appeared most frequently. The appearance of the disease-associated keywords mentioned above also increased. Therefore, we selected “frailty” for the meta-analysis (the second aim) to comprehensively evaluate the efficiency of PA on frailty, which was revealed by bibliometric analysis to be significantly increasing in recent years.

**Figure 5:**
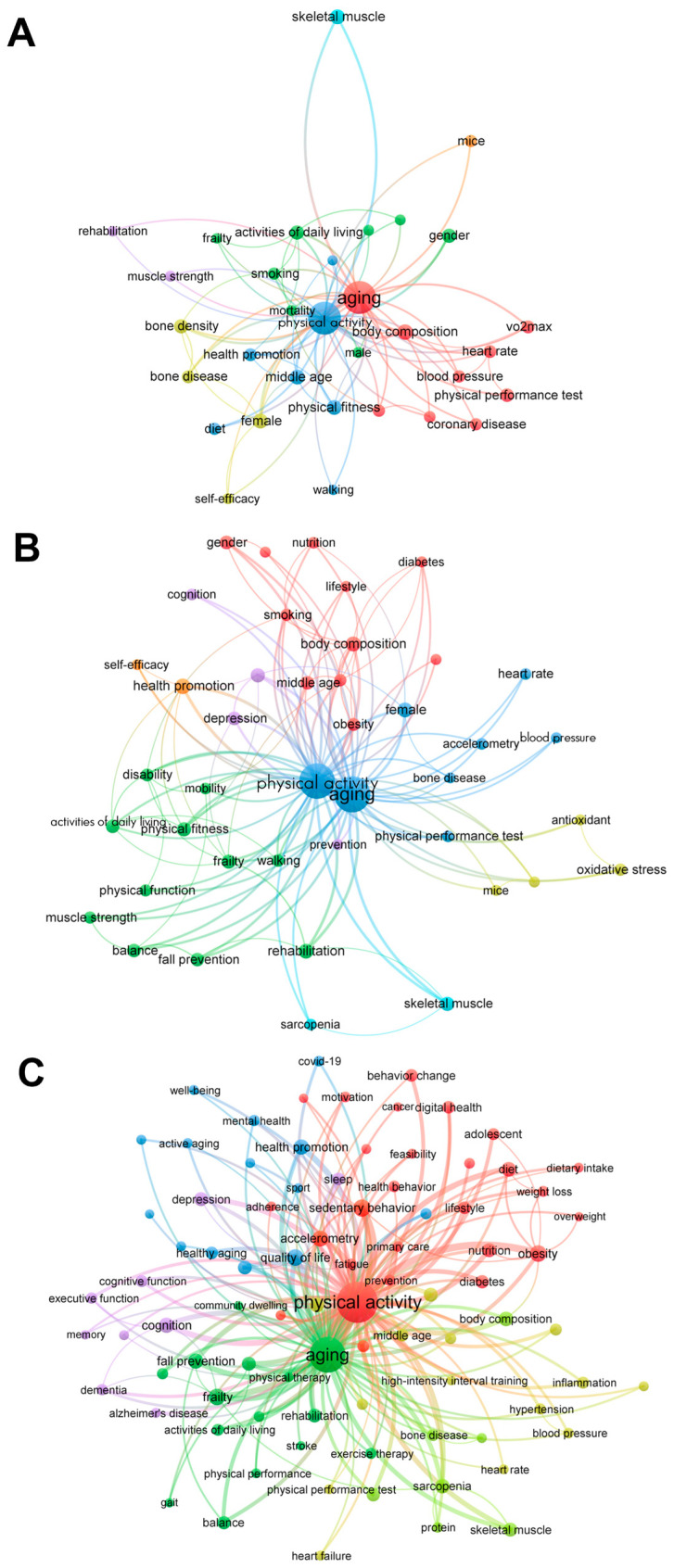
The keyword network from original articles having the two keywords “aging” and “physical activity” published between January 1991 and July 2023 classified by decade. The keyword network from original articles published between (**A**) January 1991 and December 2000 (appeared ≥7 times), (**B**) January 2001 and December 2010 (appeared ≥15 times), and (**C**) January 2011 and July 2023 (appeared ≥60 times). The similarity between two keywords is proportional to the number of simultaneous occurrences, placing words with high similarity close together. The size of the circles representing keywords increases with the relevance of the keywords (total link strength), and the distinctive colors of the circles and lines mean the difference in modularity.

Figure 5 shows the most co-occurring keywords and their distinct relationships in documents further by decade. It was found that co-occurring keywords became more diverse recently, and the number of specific keyword appearances also increased significantly, in line with the findings shown in Figure 3. A network map for the first decade composed of “body composition”, “heart rate”, and “physical performance test” suggests that the measures of body and fitness in older people and the effects of PA on them were actively examined during the first decade (Figure 5A). Consisting of newly appearing keywords “rehabilitation”, “physical fitness”, and “disability”, the network for the second decade (Figure 5B) demonstrates that PA began to be considered for its therapeutic role in improving physical function in older people during the period. Over the last decade, the network maps consisted of three keyword groups. One is a network (red) formed with “sedentary behavior”, “obesity”, and “diet”, etc., which suggests that the focus of research includes the associations between aging and PA and lifestyle and diet. Another part, a network consisting of “proteins”, “skeletal muscle”, and “sarcopenia”, suggests a focus on the biology and function of skeletal muscle in the context of aging and PA. The network consisting of other parts, such as “frailty”, “cognition”, “rehabilitation”, and “fall prevention”, shows that improvement in physical and cognitive frailty of older people due to PA and improvement in quality of life have also been hot topics recently (Figure 5C).

### 3.2. Meta-Analysis

#### 3.2.1. Quality Check

PEDro scores ranged from 4 to 8 points, with a mean score of 5.56 (±1.29). All studies fulfilled the following four criteria: random allocation, groups similar at baseline, between-group differences reported, and point estimates and variability reported. Some studies scored the criteria for assessor blinding as <15% dropout and intention-to-treat analyses. None of the studies met the criteria for participant or therapist blinding. The methodological qualities of the included studies are presented in Table 2.

#### 3.2.2. Study Characteristics

The characteristics of the 18 included studies are summarized in Table 3. The total sample size of all included studies was 1160 participants with sample sizes ranging from 22 to 115 participants, including 638 in the PA intervention group (55%) and 522 in the control group (45%). The mean age of the participants was between 70.0 (±4.7) and 93.4 (±3.2) years, and 630 were female (54%). Most studies divided participants into a control group that performed routine daily activities (n = 11 studies), although five studies provided the control group with flexibility training. RT was administered to the experimental group and mostly combined with other types of training, such as aerobic exercise, balance, gait, mobility, and flexibility training. The mean duration of the RT programs was approximately 10–12 weeks (range 8–36 weeks), and the most common training frequency was 2–3 times per week. The frailty-associated variables included in these studies were one or more of the following measurements: three body composition measurements (body mass index, muscle mass, and appendicular muscle mass), three muscular strength measurements (handgrip strength and isometric and isokinetic knee extension), four physical function measurements (one leg standing time, gait speed, timed up and go test (TUG), and short physical performance battery test (SPPB)), and two functional strength measurements (chair stand time and stair-climbing power).

#### 3.2.3. Body Composition

**Figure 6:**
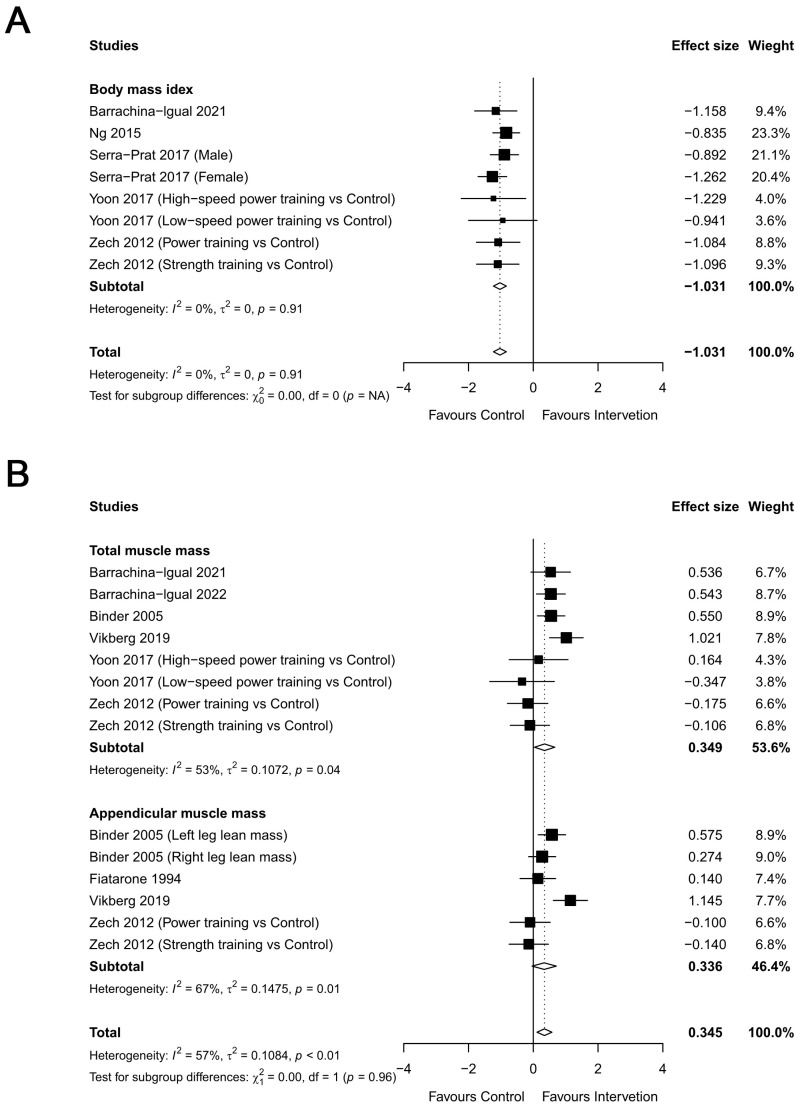
Overall meta-analysis findings and forest plot showing the comparative effect of resistance training (RT) versus the control group on body composition ((**A**) body mass index and (**B**) muscle mass) in frail elderly people. Diamonds demonstrate overall effect sizes. An effect size smaller than zero favors resistance training for body mass index. An effect size greater than zero favors RT for muscle mass [27,31,34,36,37,38,40,42,43].

Eight trials involving 452 participants provided post-intervention data on the body mass index (BMI) (Figure 6A). There was a significant improvement in BMI compared with the control with an effect size (ES) of −1.031 [95% CI:−1.230 to −0.831; *p* < 0.001]. Fourteen studies provided data on muscle masses from 779 participants (Figure 6B), showing a significant change with RT (ES = 0.345, 95% CI = 0.114 to 0.576, *p* < 0.01). Subgroup analyses were further performed, and significant RT effects were found in total muscle mass (ES = 0.349, 95% CI = 0.035 to 0.663, *p* < 0.05, I^2^ = 53%) but not in appendicular muscle mass (ES = 0.336, 95% CI = −0.038 to 0.711, *p* = 0.07, I^2^ = 67%). The heterogeneity in the results regarding body composition outcomes was very low for the body mass index (I^2^ = 0%) and moderate for muscle mass (I^2^ = 57%). These results indicate that compared with the control group, the RT group exhibited a lower BMI and increased total muscle mass but no increase in appendicular muscle mass.

#### 3.2.4. Muscular Strength

**Figure 7:**
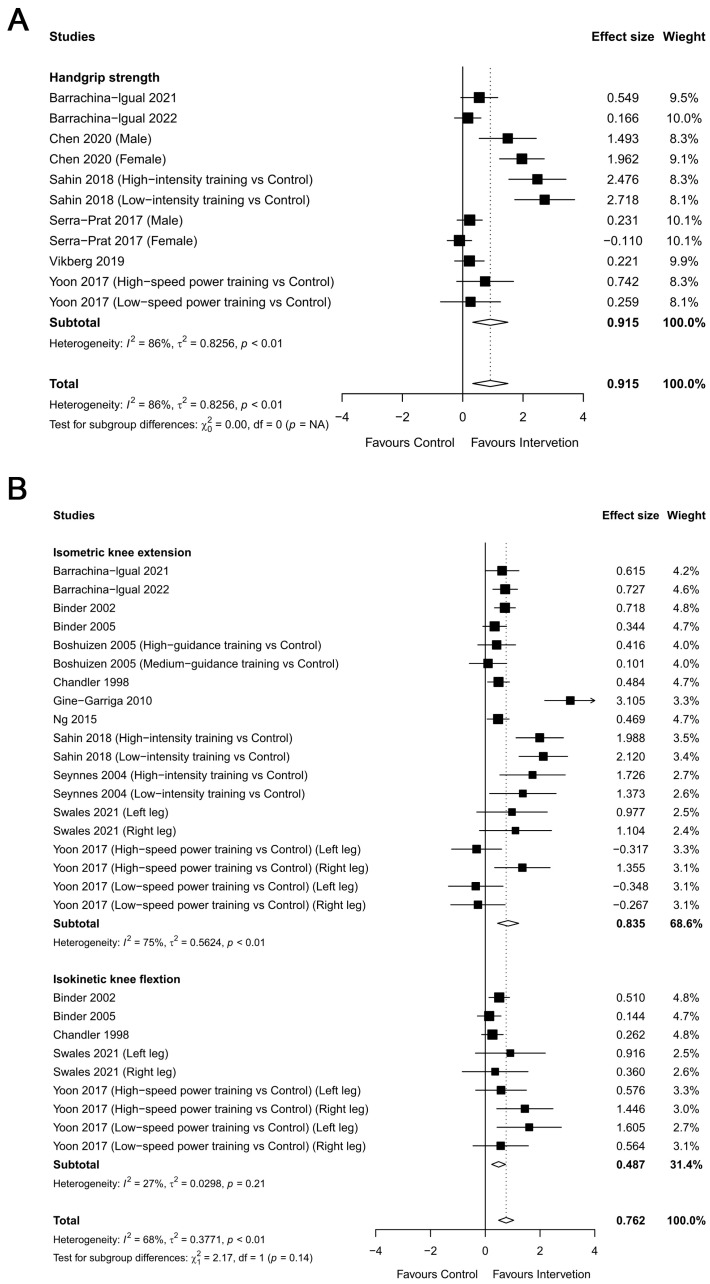
Overall meta-analysis findings and forest plot showing the comparative effect of resistance training (RT) versus the control group on muscular strength ((**A**) handgrip strength and (**B**) lower limb strength) in frail elderly people. Diamonds demonstrate overall effect sizes. Effect size greater than zero favors RT [28,29,30,31,32,33,36,37,38,39,40,41,42,43,44].

Eleven trials, including 548 participants, provided post-intervention data on handgrip strength (Figure 7A). The results demonstrate a significant difference in handgrip strength between the training group and control group (ES = 0.915, 95% CI = 0.334 to 1.500, *p* < 0.01). For lower limb strength (isometric knee extension and isokinetic knee flexion), 28 studies including 1215 participants were found (Figure 7B). The meta-analysis showed significant changes in lower limb strength (ES = 0.761, 95% CI = 0.486 to 1.04, *p* < 0.001) in the RT group. Sub-measurement analyses were performed for lower limb strength, and significant changes in both isometric knee extension (ES = 0.672, 95% CI = 0.527 to 0.818, *p* < 0.001, I^2^ = 74.7%) and isokinetic knee flexion (ES= 0.443, 95% CI= 0.239 to 0.647, *p* < 0.001, I^2^ = 26.5%) were found. The heterogeneity in the outcomes was high for handgrip strength (I^2^ = 86%) and moderate for lower limb strength (I^2^ = 68%). These results demonstrate that the RT group exhibited higher handgrip strength and lower limb muscle strength (isometric knee extension and isokinetic knee flexion), compared with the control group.

#### 3.2.5. Physical Function

**Figure 8:**
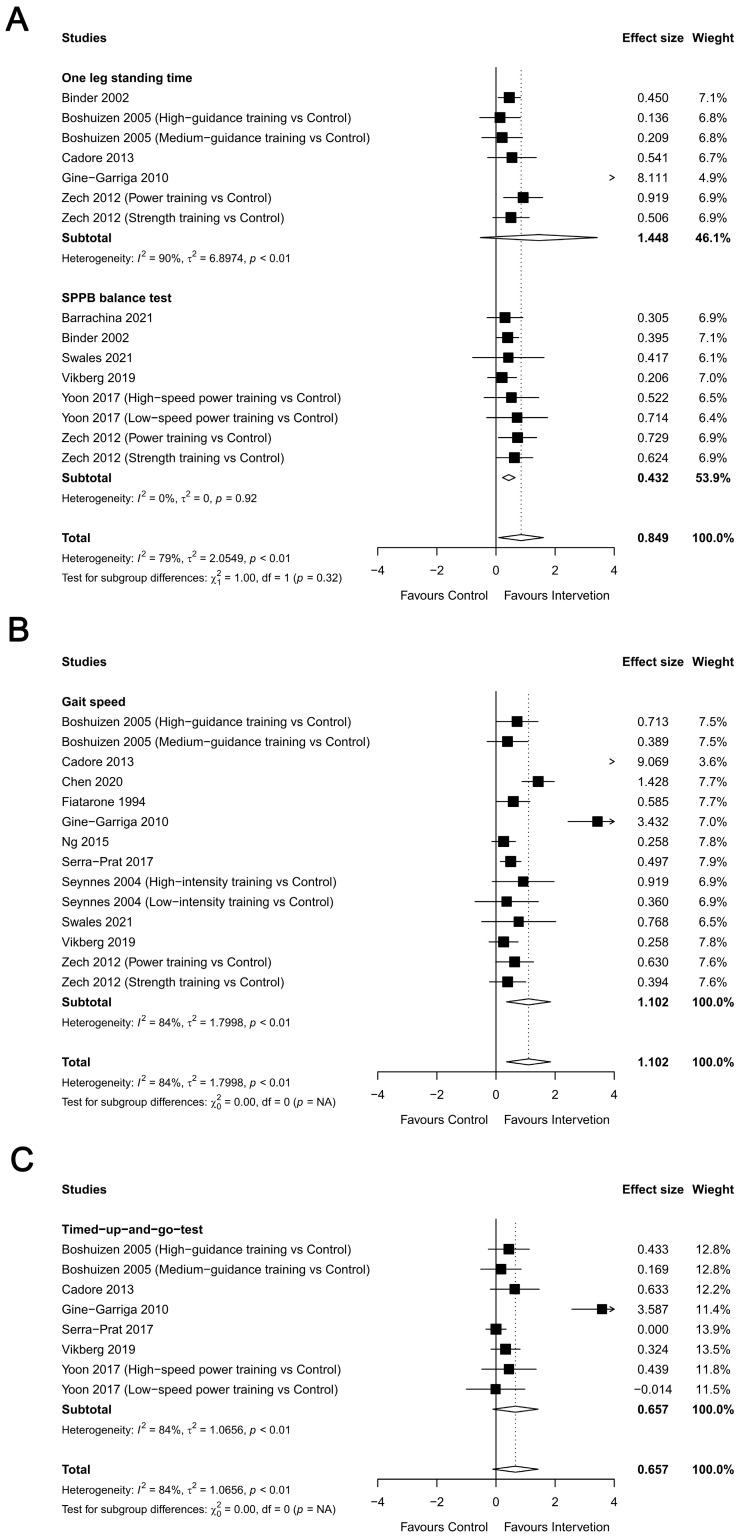
Overall meta-analysis findings and forest plots showing the comparative effect of resistance training (RT) versus the control group on the physical function ((**A**) balance (one leg standing, short physical performance battery (SPPB)), (**B**) gait speed, and (**C**) timed up and go test (TUG)) of frail elderly people. Diamonds demonstrate overall effect sizes. Effect size greater than zero favors RT [27,29,30,32,33,34,35,36,37,38,40,41,42,44].

The effects of RT were reported for balance in 15 studies (681 participants), gait speed in 14 studies (667 participants), and agility (TUG) in eight studies (366 participants). The meta-analysis found significant changes in favor of RT group for balance (ES = 0.849, 95% CI = 0.093 to 1.61, *p* < 0.05) (Figure 8A) and gait speed (ES = 1.101, 95% CI = 0.357 to 1.85, *p* < 0.05) (Figure 8B); however, there was no significant RT effect on agility (ES = 0.657, 95% CI = −0.107 to 1.422, *p* = 0.092) (Figure 8C). Sub-measurement analyses for balance yielded a positive significant effect on one-leg standing time (ES = 1.448, 95% CI = −0.529 to 3.425, *p* < 0.01, I^2^ = 90%) and the SPPB balance test (ES = 0.432, 95% CI = 0.219 to 0.646, *p* < 0.001, I^2^ = 0%). The heterogeneity in the results around these outcomes was moderate for balance (I^2^ = 60.5%) and high for gait speed (I^2^ = 83.6%) and agility (I^2^ = 84.1%). In summary, RT demonstrated significant improvements in balance and gait speed but no significant effect on agility.

#### 3.2.6. Functional Strength

**Figure 9:**
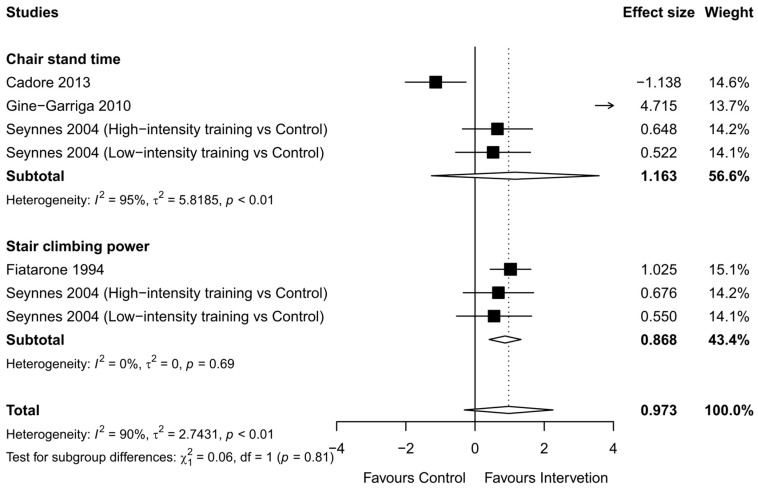
Overall meta-analysis findings and forest plots showing the comparative effect of resistance training (RT) versus the control group on functional strength (chair stand and stair climbing) in frail elderly people. Diamonds demonstrate overall effect sizes. An effect size greater than zero favors RT [27,30,33,35].

Seven trials involving 176 participants provided post-intervention data on functional strength (Figure 9). There was not a significant improvement in functional strength compared with the control after pooling the results (ES = 0.973, 95% CI = −0.311 to 2.256, *p* = 0.09). As a result of the sub-measurement analyses, the chair stand test results, reported in four studies (95 participants), did not change with RT (ES = 1.163, 95% CI = −1.260 to 3.585, *p* = 0.32). A significant change was observed in the stair-climbing test (ES = 0.870, 95% CI = 0.410 to 1.330, *p* < 0.001). The heterogeneity in the results around the outcomes was high for the total functional strength (I^2^ = 90%) and chair stand test (I^2^ = 95%), but very low for the stair-climbing test (I^2^ = 0%). Thus, RT did not show a significant improvement in overall functional strength compared with the control, although RT did improve stair-climbing ability.

## 4. Discussion

By 2050, the number of older adults is expected to almost double to 2.1 billion because of increases in life expectancy [52]. Nevertheless, an increase in life expectancy may not convert into an increase in lifespan without disability, and these individuals may experience poor general health during their prolonged years [7]. Therefore, maintaining the physical health of older adults is a critical public health concern. Accumulating evidence indicates that PA is a highly effective non-therapeutic approach for promoting healthy aging [14,15]. PA can improve functional capabilities and mitigate physical comorbidities associated with aging [16], directly contributing to quality of life. Therefore, efforts to elucidate the roles of PA in healthy aging have accumulated over the past few decades [19]. However, there have been no attempts to identify the large volumes and growth patterns in the accumulated research data affecting the development of aging and PA research, and no bibliometric analysis has examined the connection between them. Accordingly, the first aim of this study was to review the literature published on aging and PA research using a bibliometric analysis to illustrate the research landscape and identify hot topics and emerging trends in aging and PA research.

The results of the bibliometric analysis show that a handful of publications between the 1990s and the 2000s have increased to a substantial research field in recent years. This trend reflects the growing interest of institutions and researchers in aging and PA, highlighting their essential roles in human disease, health, and lifespan. These results are meaningful in that this is the first attempt, to our knowledge, to explain and visualize intuitively the research trends in the role of PA in aging, emphasizing that PA is essential for healthy aging. Findings from the author keyword visualization map demonstrate that overall, co-occurring keywords with aging and PA can be categorized into three subject categories: (1) physical function and rehabilitation, (2) lifestyle factors, and (3) cognitive function. In addition, the research trend classified by era indicates that while studies from the early (1990s) to mid-term (2000s) periods mainly investigated the relationship between lifestyle factors, aging, and PA, more recently, studies have been conducted with a focus on specific aging-associated diseases, such as frailty, sarcopenia, and depression. Considering the current research gaps in our knowledge of the underlying mechanisms of many chronic diseases and the fact that aging is highly associated with the diseases, our findings suggest that exploring the mechanisms by which aging contributes to these diseases and how PA can prevent or ameliorate the effects of aging would be highly demanded and warranted as the future research agenda.

The bibliometric analysis revealed that “frailty” is the fastest-growing research keyword in the fields of aging and PA research. This is consistent with recent studies highlighting that frailty affects an estimated 11% of older adults [53] and is the most common condition influencing older adults in terms of both mortality and morbidity [54]. Furthermore, the recent generations of older adults tend to have higher frailty levels [55]. Therefore, the second aim of this study was to investigate the effects of RT, the recommended PA type for older people [24], on frail older individuals using a meta-analysis of the literature that studied the effects of RT on the body composition, muscular strength, and physical function of frail older adults. The results of this meta-analysis provide evidence that RT is effective in lowering BMI, increasing muscle mass, and improving muscle strength, balance, and walking speed. Our study is not only the first to apply bibliometric analysis to aging and PA research but also the first to logically verify the effect of PA (RT) using a meta-analysis targeting the main keyword found with bibliometric analysis.

The results from our meta-analysis demonstrated a significant reduction in BMI among frail older adults subjected to RT. This suggests a potential avenue for improving frailty by lowering BMI, which is supported by earlier research linking a heightened frailty risk to overweight and obese states based on BMI [56]. However, it is also notable that BMI does not directly indicate body composition such as adiposity, which becomes more pronounced with age and is associated with progression to sarcopenic obesity [57,58]. Additionally, women may be more susceptible to frailty due to their higher intrinsic adiposity [59], and older women are more likely to experience obesity-related frailty [60]. Thus, it is important to acknowledge the limitations of associating BMI with total frailty across sexes.

A result of this meta-analysis revealed a significant increase in muscle mass in response to RT in frail older adults as expected. A reduction in lean body mass with an accompanying increase in fat mass is one of the most striking and consistent changes observed with aging [3]. Low muscle mass is considered an inevitable condition and a key component of physical frailty [61]. Increasing muscle mass in older adults can be challenging because age-related changes in hormonal profiles and physiological functions can hinder muscle protein synthesis [62]. However, studies have shown that RT can effectively increase muscle mass in older adults. This beneficial effect of RT on muscle mass has also been observed in individuals with other health conditions and functional limitations [63]. Furthermore, in older adults, these beneficial changes in body composition characteristics with RT can lower the risk of other common disorders such as metabolic syndrome and diabetes [64]. Muscular strength, which is directly related to muscle mass, is a crucial component of physical function and is associated with various health outcomes in older adults [65]. Handgrip and leg strength tests are widely used to measure muscle strength [66]. We assessed the effects of RT on these two measures. Handgrip strength is often used as an indicator of overall muscle strength in aging adults and physical function in older adults, and low handgrip strength is associated with a variety of poor health outcomes, including chronic morbidities, functional disabilities, and all-cause mortality [67]. Similarly, lower limb strength is critical for maintaining mobility and independence in older adults [68]. In line with previous studies, our results demonstrate that RT has a significant positive effect on both handgrip strength and lower limb strength, indicating that RT helps enhance or preserve muscular strength and thus prevents or ameliorates frailty in older adults.

Loss of mobility is especially problematic since it has a significant negative influence on quality of life and is strongly linked to poor health outcomes, disability, and loss of independence [69]. Age-related losses in balance and gait are observed in older adults, who also exhibit increased gait variability and a corresponding rise in fall risk [70]. These are particularly important given the importance of measures for fall prevention and overall mobility in older adults [71]. Our results demonstrate that RT is an effective way to improve balance and gait speed, which has implications for maintaining independence and quality of life in older adults. The TUG test has been extensively used to assess balance and mobility simultaneously in older adults [72,73,74], and previous studies have shown that RT improves TUG test scores in healthy older adults [75]. However, our meta-analysis found no significant effect of RT on TUG test scores. This could be due to the small number of studies (n = 8) and/or heterogeneity in the RT program, suggesting the necessity for further studies to investigate the effects of RT on the TUG test in older adults. In addition, our findings indicated that RT had no significant effect on chair stand time. One possible explanation could be the high heterogeneity observed in the trials, as indicated by the high standard deviation (Tau2 = 5.82) and small number of studies (n = 4). Future studies are warranted to examine the effects of RT on the chair stand test in frail older adults to confirm the results of previous studies. In contrast, a significant improvement in stair climbing was observed among individuals who participated in the RT programs. This finding is consistent with previous studies showing that RT can improve lower limb muscle power, which is critical for tasks such as stair climbing [76].

Combining the effects of RT on various measurements from the meta-analysis, our results demonstrate that RT counteracts or postpones aging-associated declines in intrinsic capacity (body composition, muscle strength, and physical function). Therefore, it can be concluded that PA is important for healthy aging.

## 5. Conclusions

Our study is the first to analyze research trends in aging and PA studies using a bibliometric analysis with a follow-up meta-analysis with a focus on frailty, which was found to be the most popular co-occurring keyword with aging and PA. The bibliometric analysis revealed that the number of publications on this research topic has increased steadily from the 1990s to the present, indicating a growing interest in understanding the role of PA in aging and its importance in human health. Frailty was found to be the most noteworthy keyword co-occurring with aging and PA. Thus, we further investigated the effects of RT on frail older adults. The meta-analysis found that RT had significant positive effects on physical factors associated with frailty, including handgrip strength, lower limb strength, balance, gait speed, and stair-climbing ability in frail older individuals, with few exceptions such as the TUG or chair stand time tests. These findings indicate that RT is an effective intervention for improving physical function among frail older adults, particularly for tasks that require lower limb muscle strength.

Given that the global population is aging, our results from the bibliometric analysis provide summarized and visualized evidence that PA has become a field of great interest in the association with aging. In particular, our meta-analysis findings have important implications for the development of PA programs that can help older adults maintain their independence and quality of life by alleviating physical frailty. These emphasize that PA is essential for healthy aging. Future research should investigate the optimal dose, frequency, and duration of RT programs for older adults, as well as the potential benefits of combining RT with other forms of PA, such as aerobic or balance exercises.

## Figures and Tables

**Table 1:** Top 30 co-occurring keywords with aging and physical activity by decade.

	1991–2000			2001–2010			2011–August 2023		
	Keyword	Occurrences	Total Link Strength	Keyword	Occurrences	Total Link Strength	Keyword	Occurrences	Total Link Strength
1	body composition	29	77	body composition	50	145	sedentary behavior	485	1290
2	female	25	68	health promotion	58	136	obesity	447	1225
3	physical fitness	24	68	rehabilitation	48	116	quality of life	443	1108
4	middle age	23	57	quality of life	41	115	frailty	424	1126
5	activities of daily living	19	56	female	46	113	accelerometry	356	951
6	bone density	19	51	frailty	43	103	cognition	329	899
7	gender	19	47	physical fitness	42	98	health promotion	326	902
8	skeletal muscle	23	45	depression	33	96	fall prevention	311	820
9	health promotion	15	45	obesity	31	92	physical fitness	283	760
10	bone disease	15	43	skeletal muscle	40	91	diet	279	807
11	smoking	13	43	gender	38	90	body composition	265	668
12	heart rate	15	38	fall prevention	37	90	sarcopenia	260	719
13	VO_2_ max	15	35	balance	33	83	physical function	256	688
14	cardiovascular disease	11	35	disability	29	82	depression	253	653
15	risk factor	10	35	diet	26	81	rehabilitation	252	619
16	diet	13	31	smoking	23	75	skeletal muscle	245	602
17	blood pressure	11	31	middle age	33	70	nutrition	228	666
18	coronary disease	11	28	mobility	20	68	walking	227	607
19	longitudinal study	8	27	activities of daily living	33	67	female	223	520
20	mortality	8	27	walking	27	64	diabetes	212	523
21	male	8	24	muscle strength	26	62	muscle strength	203	524
22	physical performance test	16	23	physical function	26	59	balance	192	500
23	mice	12	23	nutrition	19	58	middle age	182	360
24	muscle strength	8	23	oxidative stress	23	51	healthy aging	178	400
25	leisure activity	8	21	cognition	20	50	lifestyle	176	546
26	self-efficacy	8	17	lifestyle	18	50	sleep	166	445
27	frailty	7	17	sarcopenia	21	47	digital health	157	471
28	walking	7	16	self-efficacy	17	47	behavior change	156	412
29	questionnaire	7	16	diabetes	17	46	adolescent	152	369
30	rehabilitation	7	13	prevention	16	43	activities of daily living	143	392

**Table 2:** Physiotherapy Evidence Database (PEDro) scale of the studies included in the meta-analysis.

Study	1	2	3	4	5	6	7	8	9	10	11	PEDro Score (0–10)
Fiatarone et al., 1994 [27]	Y	Y	N	Y	N	N	Y	Y	Y	Y	Y	7
Chandler et al., 1998 [28]	Y	Y	N	Y	N	N	Y	Y	N	Y	Y	6
Binder et al., 2002 [29]	Y	Y	N	Y	N	N	Y	N	N	Y	Y	5
Seynnes et al., 2004 [30]	Y	Y	N	Y	N	N	N	N	N	Y	Y	4
Binder et al., 2005 [31]	Y	Y	N	Y	N	N	N	N	N	Y	Y	4
Boshuizen et al., 2005 [32]	Y	Y	N	Y	N	N	N	N	N	Y	Y	4
Giné-Garriga et al., 2010 [33]	Y	Y	Y	Y	N	N	Y	N	N	Y	Y	6
Zech et al., 2012 [34]	Y	Y	Y	Y	N	N	Y	Y	N	Y	Y	7
Cadore et al., 2014 [35]	Y	Y	Y	Y	N	N	Y	N	N	Y	Y	6
Ng et al., 2015 [36]	Y	Y	Y	Y	N	N	Y	Y	Y	Y	Y	8
Serra-Prat et al., 2017 [37]	Y	Y	Y	Y	N	N	N	N	N	Y	Y	5
Yoon et al., 2017 [38]	Y	Y	N	Y	N	N	N	N	N	Y	Y	4
Sahin et al., 2018 [39]	Y	Y	N	Y	N	N	N	N	N	Y	Y	4
Vikberg et al., 2019 [40]	Y	Y	Y	Y	N	N	Y	Y	N	Y	Y	7
Chen et al., 2020 [41]	Y	Y	Y	Y	N	N	Y	Y	N	Y	Y	7
Barrachina-Igual et al., 2021 [42]	Y	Y	N	Y	N	N	N	Y	N	Y	Y	5
Barrachina-Igual et al., 2022 [43]	Y	Y	N	Y	N	N	Y	N	N	Y	Y	5
Swales et al., 2022 [44]	Y	Y	N	Y	N	N	N	Y	Y	Y	Y	6

N, no, does not meet the criteria; Y, yes, meets the criteria. Eligibility criteria items do not contribute to the total score. The PEDro scale criteria are (1) eligibility criteria; (2) random allocation; (3) concealed allocation; (4) groups similar at baseline; (5) blinding of participants; (6) blinding of therapists; (7) blinding of assessors; (8) adequate follow-up (<15% dropouts); (9) intention-to-treat analysis; (10) between-group comparisons; and (11) point estimates and variability.

**Table 3:** Characteristics of the included studies for meta-analysis.

Study (Year, Country)	N (IG/CG)	Age (Years)	Gender (M/F)	Duration (Frequency)	Intervention	CG
Fiatarone et al. 1994 (USA) [27]	51 (25/26)	IG 86.2 ± 5.0 CG 89.2 ± 4.0	21/30	10 (3×/week)	**Progressive lower extremity RT** - Hip and knee extensors (80% of 1-RM).	-
Chandler et al. 1998 (USA) [28]	100 (50/50)	77.6 ± 7.6	50/50	10 (3×/week)	**Progressive lower extremity RT** - Resisted hip extension and abduction, knee flexion and extension, ankle dorsiflexion, toe raises, chair rises, and stair stepping (two sets × 10 reps). - Once the participant could perform two sets of 10 easily at a given color of theraband, the resistance was increased by replacing the theraband with the next color.	No RT was allowed, but aerobic or flexibility exercises were permitted.
Binder et al. 2002 (USA) [29]	115 (66/49)	IG 83.0 ± 4.0 CG 83.0 ± 4.0	55/60	36 (2–3×/week)	**Three approximately 3-month-long phases of ET** - Phase 1: twenty-two exercises that focused on improving flexibility, balance, coordination, speed of reaction, and, to a modest extent, strength. - Phase 2: progressive RT (one to two sets × six to eight reps, 65% of 1 RM). - Phase 3: endurance training 15–20 min (65–75% of VO_2_peak). - Shortened programs of Phase 1 and Phase 2 exercises were continued during Phase 3.	Home-based exercise program focused primarily on flexibility (60 min, 2–3×/week) was performed.
Seynnes et al. 2004 (France) [30]	22 (HI 8/LI 6/CG 8)	81.5	N/A	10 (3×/week)	**Progressive lower extremity RT** - Knee extensor muscles. - HI: Three sets × eight reps, 80% of 1 RM. - LI: Three sets × eight reps, 40% of 1 RM.	-
Binder et al. 2005 (USA) [31]	91 (53/38)	83.0 ± 4.0	42/49	36 (3×/week)	**Multi-component exercise program** - Weeks 1–12: light-resistance + flexibility + balance training. - Weeks 13–24: resistance + flexibility + balance training. - Weeks 25–36: resistance + flexibility + balance + endurance training.	Home-based exercise program focused primarily on flexibility (60 min, 2–3×/week) was performed.
Boshuizen et al. 2005 (Netherlands) [32]	72 (HG 24/MG 26/CG 22)	HG 80.0 ± 6.7 MG 79.3 ± 7.0 CG 77.2 ± 6.5	4/68	10 (3×/week)	**Lower extremity RT** - HG: two group sessions + one home session. - MG: one group session + two home session.	-
Giné-Garriga et al. 2010 (Spain) [33]	51 (26/25)	84.0 ± 2.9	20/31	12 (2×/week)	**Progressive lower extremity RT + balance training** - One day of balance-based activities and one day of lower body strength-based exercises (RPE intensity of 12–14; one to two sets × six to eight reps).	-
Zech et al. 2012 (Germany) [34]	69 (ST 23/PT 24/CG 22)	ST 77.8 ± 6.1 PT 77.4 ± 6.2 CG 75.9 ± 7.8	N/A	12 (2×/week)	**Progressive RT + balance training** - ST: with an “average” velocity (2–3 s). - PT: move as rapidly as possible during the concentric phase of each repetition and move slowly during the eccentric phase (2–3 s).	-
Cadore et al. 2014 (Spain) [35]	24 (11/13)	IG 93.4 ± 3.2 CG 90.1 ± 1.1	7/14	12 (2×/week)	**Multi-component exercise program** - Upper and lower body RT with progressively increased loads (1 × 8^−10^ reps, 40–60% of 1 RM) with balance and gait retraining.	Mobility exercises were performed 30 min per day (4×/week).
Ng et al. 2015 (Singapore) [36]	98 (48/50)	70.0 ± 4.7	43/55	24 (2×/week)	**Progressive RT + functional tasks + balance training** - Weeks 1–12: classes conducted by a qualified trainer. - Weeks 13–24: home-based exercises.	-
Serra-Prat et al. 2017 (Spain) [37]	172 (80/92)	IG 77.9 ± 5.0 CG 78.8 ± 4.9	75/97	48 (4×/week)	**Multi-component exercise program** - Aerobic exercise: walking outdoors for 30–45 min. - Strengthening arms, legs, and balance training for 20–25 min.	-
Yoon et al. 2017 (Korea) [38]	58 (HSPT 19/LSST 19/CG 20)	HSPT 75.0 ± 0.9 LSST 76.0 ± 1.3 CG 78.0 ± 1.0	0/58	12 (2×/week)	**Progressive RT with elastic bands** - HSPT: low intensity, two to three sets × 12–15 reps. - LSST: high intensity, two to three sets × 8–10 reps.	Static and dynamic stretching exercises (60 min, 1×/week) were performed.
Sahin et al. 2018 (Turkey) [39]	48 (HI 16/LI 16/CG 16)	HI 84.2 ± 6.9 LI 84.5± 4.8 CG 85.4 ± 4.7	N/A	8 (3×/week)	**Whole body RT** - HI: one set × 6–10 reps, 70% of 1 RM. - LI: one set × 6–10 reps, 40% of 1 RM.	-
Vikberg et al. 2019 (Sweden) [40]	70 (36/34)	70.9 ± 0.03	32/38	10 (3×/week)	**Whole body RT, with a focus on strengthening of the lower-extremity** - Moderate to high RT intensity was applied using the Borg CR-10 scale (6–7/10). - Week 1: two sets × 12 reps. - Weeks 2–4: three sets × 10 reps. - Weeks 5–7: four sets × 10 reps. - Weeks 8–10: four sets × 10 reps.	-
Chen et al. 2020 (China) [41]	70 (35/35)	IG 77.0 ± 5.2 CG 75.3 ± 6.0	23/43	8 (3×/week)	**Whole body RT with elastic bands** - Two sets × 10^−15^ reps.	-
Barrachina-Igual et al. 2021 (Spain) [42]	50 (27/23)	75.0 ± 7.0	13/37	12 (2×/week)	**Multi-component exercise program** - Balance, flexibility, aerobic (10 min warming-up), and progressive RT (three sets × 10–15 reps, 70% of 1 RM) combined with self-massage for myofascial release.	-
Barrachina-Igual et al. 2022 (Spain) [43]	81 (39/42)	77.6 ± 7.5	13/68	20 (2×/week)	**Multi-component exercise program** - Warm-up exercise: aerobic exercise and joint mobility for 10 min. - Progressive high-intensity RT: six strength exercises (two trunk, two arms, and two legs) three sets × 8–12 rep for 42–45 min. - Self-massage for myofascial release: seven exercises per session (four lower limb, one chest, and two back) one set × 10 reps for 9–10 min.	Continue their routine daily activities.
Swales et al. 2022 (UK) [44]	11 (6/5)	86.1 ± 7.2	4/7	6 (3×/week)	**RT** - Warm-up for 5 min. - Resistance training: optimal rhomboid, hip adduction, hip abduction, chest press, leg extension, leg curl, leg press (two sets × 12 reps).	-

IG, intervention group; CG, control group; M, male; F, female; RT, resistance training, RM, repetition maximum; HG, high-guidance group; MG, medium-guidance group; RPE, rating of perceived exertion; ST, strength training; PT, power training; HSPT, high-speed power training; LSST, low-speed strength training; HI, high-intensity RT; LI, low-intensity RT. Data for age are mean ± standard deviation.

## Data Availability

Data are contained within the article.
